# Renal resistive index as an early predictor and discriminator of acute kidney injury in critically ill patients; A prospective observational cohort study

**DOI:** 10.1371/journal.pone.0197967

**Published:** 2018-06-11

**Authors:** Jelle L. G. Haitsma Mulier, Sander Rozemeijer, Jantine G. Röttgering, Angelique M. E. Spoelstra-de Man, Paul W. G. Elbers, Pieter Roel Tuinman, Monique C. de Waard, Heleen M. Oudemans-van Straaten

**Affiliations:** Department of Intensive Care Medicine, VU University Medical Center, Amsterdam, The Netherlands; University of Sao Paulo Medical School, BRAZIL

## Abstract

**Background:**

Acute kidney injury (AKI) complicates shock. Diagnosis is based on rising creatinine, a late phenomenon. Intrarenal vasoconstriction occurs earlier. Measuring flow resistance in the renal circulation, Renal Resistive Index (RRI), could become part of vital organ function assessment using Doppler ultrasound. Our aim was to determine whether RRI on ICU admission is an early predictor and discriminator of AKI developed within the first week.

**Methods:**

In this prospective cohort of mixed ICU patients with and without shock, RRI was measured <24-h of admission. Besides routine variables, sublingual microcirculation and bioelectrical impedance were measured. AKI was defined by the Kidney Disease Improving Global Outcomes criteria. Uni- and multivariate regression and Receiver Operating Characteristics curve analyses were performed.

**Results:**

Ninety-nine patients were included, median age 67 years (IQR 59–75), APACHE III score 67 (IQR 53–89). Forty-nine patients (49%) developed AKI within the first week. AKI patients had a higher RRI on admission than those without: 0.71 (0.69–0.73) vs. 0.65 (0.63–0.68), p = 0.001. The difference was significant for AKI stage 2: RRI = 0.72 (0.65–0.80) and 3: RRI = 0.74 (0.67–0.81), but not for AKI stage 1: RRI = 0.67 (0.61–0.74). On univariate analysis, RRI significantly predicted AKI 2–3: OR 1.012 (1.006–1.019); Area Under the Curve (AUC) of RRI for AKI 2–3 was 0.72 (0.61–0.83), optimal cut-off 0.74, sensitivity 53% and specificity 87%. On multivariate analysis, RRI remained significant, independent of APACHE III and fluid balance; adjusted OR: 1.008 (1.000–1.016).

**Conclusions:**

High RRI on ICU admission was a significant predictor for development of AKI stage 2–3 during the first week. High RRI can be used as an early warning signal RRI, because of its high specificity. A combined score including RRI, APACHE III and fluid balance improved AKI prediction, suggesting that vasoconstriction or poor vascular compliance, severity of disease and positive fluid balance independently contribute to AKI development.

**Trial registration:**

ClinicalTrials.gov NCT02558166.

## Introduction

Acute kidney injury (AKI) occurs as a serious complication of septic or cardiogenic shock and major surgery in 30 to 57% of critically ill intensive care patients. In its severe form, AKI requires renal replacement therapy, which is applied in 5–13% of ICU patients [1,2]. Since AKI increases morbidity and mortality, early detection and prevention are crucial [3,4].

Mechanisms of AKI comprise renal hypoperfusion, intrarenal vasoconstriction, inflammation, oxidative stress and nephrotoxicity [5]. An important pathophysiological pathway includes intrarenal vasoconstriction and endothelial damage of the microvessels, leading to impaired macro- and microvascular flow, which further aggravates ischemia [6]. Monitoring blood pressure and cardiac output are part of clinical practice to titrate the administration of fluids and vasoactive drugs in patients with compromised circulation and shock. However, the monitoring of kidney perfusion is not daily practice yet.

Nowadays Doppler ultrasound is rapidly gaining ground as a screening tool in critically ill patients. The performance of cardiac, lung and abdominal ultrasound in patients after cardiac arrest, major operations and during shock has become standard policy. However, renal ultrasound, which could be easily incorporated in this screening, is not commonly performed. Renal vasoconstriction is an early manifestation of AKI. Renal Doppler ultrasound can measure the renal resistive index (RRI), a sonographic index that reflects alterations in blood flow profile of the intrarenal arcuate or interlobar arteries. It reflects the relation between the decline in speed loss of flow (“flow velocity”) between the peak of systole and the end of diastole in (renal) blood vessels: RRI = (peak systolic velocity—end diastolic velocity)/(peak systolic velocity). Normal values are reported between 0.60 and 0.70 with the difference between both kidneys mostly being less than 5% [7–9].

Previous studies have shown that elevated RRI is related to hemodynamic parameters such as systolic- and diastolic blood pressure, pulse pressure and pulse wave velocity, which is a measure for arterial stiffness [10–13]. Furthermore several diseases such as atherosclerosis, diabetes (even in the pre-micro-albuminuric phase), chronic kidney disease and histopathological outcomes (glomerular sclerosis, arteriolosclerosis, interstitial fibrosis), are associated with elevated RRI [8,10,14–17]. RRI is significantly related to endpoints such as the degree of microalbuminuria [18]. Also several pharmacological factors influence RRI. For example ACE-inhibitor treatment in patients with primary hypertension has been associated with a decrease in RRI [19] while norepinephrine dose correlated with higher RRI [20], However, epinephrine and dopamine dose were not related with RRI [21].

Data on the predictive value of RRI for the development of AKI, independent of other risk factors, are controversial [22–24]. Furthermore, none of the previous studies on the predictive value of RRI for AKI accounted for the sublingual microcirculation, fluid balance and hydration as independent risk factors.

The present study questions the clinical relevance of the RRI in terms of AKI prediction in the light of microcirculation, fluid balance and other risk factors. The primary aim was to determine whether the RRI on ICU admission predicts the development of AKI during the first week. Secondary aims were to determine whether RRI is related to the severity of AKI and whether the RRI on ICU admission predicts the development of AKI independent of other known risk factors including severity of disease, microcirculatory markers, fluid balance and hydration.

## Methods

This observational cohort study was performed in the 24-bed mixed medical and surgical intensive care unit of the VU university medical center in Amsterdam, The Netherlands. This study is part of a larger project including the study of determinants of the RRI. Approval to conduct this study was obtained from our institution’s independent review committee (Record METC-2015.025). The committee agreed with a deferred consent procedure for the use of data to be obtained from the surviving patients if awake and able to communicate or from their legal representative. To avoid inclusion bias, the board agreed with the use of data for analysis from the patients who had died or patients who were unable to give deferred consent due to neurological damage or severe mental instability [25,26]. Sample size calculations were based on the assumption that patients not developing AKI would have an RRI of 0.72 and patients developing AKI would have an AKI of 0.79 with a standard deviation of 0.11. These calculations were based on the results of 3 previous studies that reported median values for AKI and no AKI [22,27,28]. To provide a power of 80% with an α of 0.05, we had to include a total of 39 patients developing AKI and 39 patients not developing AKI. To increase the hazard of AKI we decided to include half of the patients with shock. Inclusion criteria were: age >18 years and inclusion within 24 hours after ICU admission. Exclusion criteria were: poor abdominal echogenicity, severe acute or chronic renal insufficiency defined as eGFR < 30 ml/min/1.73m^2^, dialysis dependency, renal transplantation, known renal artery stenosis, pregnancy, mono-kidney, kidney tumor, anatomic kidney abnormalities or suicide attempt.

### Definitions

AKI was defined according to the Kidney Disease Improving Global Outcome (KDIGO) classification using both creatinine and urine output criteria [29]. As presented in Table 1, KDIGO distinguishes three stages of renal dysfunction: AKI stage 1, 2 and 3. Patients were evaluated for AKI daily for 7 days after inclusion. If a patient met the criteria at any moment of this 7-day period, he was considered as having AKI. Shock was defined as persistent hypotension requiring vasopressor therapy after adequate fluid resuscitation in the presence of perfusion abnormalities, manifest by poor peripheral perfusion, organ dysfunction or lactate > 2 mmol/L [30]. Patients who only needed a low short-term vasopressor dose during propofol sedation without signs of perfusion abnormalities were not considered as being in shock. Cardiogenic shock was defined as circulatory shock caused by heart failure documented by poor contractility on ultrasound or low cardiac index (CI <2L/min) after fluid resuscitation. Cardiac ultrasound was part of the routine work-up in all patients needing vasopressors who did not have a pulmonary artery catheter. Hypovolemic shock was defined as shock due to excessive fluid loss, requiring fluid resuscitation and vasopressors. Septic shock was defined as shock caused by proven or suspected infection [31]. Non-shock was defined as: fluid- and vasopressor- independent circulation. Severity of shock was quantified by the duration and dose of vasopressors.

**Table 1:** KDIGO AKI classification.

Stage	Serum creatinine	Urine output
1	1.5–1.9 times baseline OR ≥ 0.3 mg/dL (≥ 26.5μmol/L) increase	<0.5ml/kg/h for 6–12 hours
2	2.0–2.9 times baseline	<0.5ml/kg/h for ≥ 12 hours
3	3.0 times baseline OR Increase in serum creatinine to ≥ 4.0mg/dL (≥ 353.9 μmol/L) OR Initiation of renal replacement therapy OR, in patients <18 years, decrease in eGFR to < 35 mL/min per 1.73m^2^	<0.3ml/kg/h for ≥ 24 hours OR Anuria for ≥ 12 hours

### Measurements

Study measurements were performed within 24 hours after ICU admission within the timespan of one hour as soon as possible after stabilizing the patient. *Renal resistive index* was measured with ultrasound-Doppler using a transparietal 5MHz pulsed-wave Doppler probe (C5-1 ultrasound transducer, Philips Medical Systems International B.V., Best, The Netherlands) on a CX50 ultrasound system by two independent, trained sonographers (J.H.M. and S.R.) who were not involved in patient care. After visualizing the kidney in ultrasound mode and checking for renal abnormalities, an arcuate or interlobar artery was localized and three successive Doppler measurements at different positions in the kidney (high, middle and low) were performed, 3 times in each kidney. So a total number of 9 RRI values were obtained in each kidney. The median value of each section was used and the 3 median values of each kidney were averaged. *Sublingual microcirculation* was measured using Sidestream Dark Field imaging (SDF) [32]. When possible, patients were measured at 3–5 different sublingual sites. Videos of these measurements were recorded and analyzed afterwards by an independent researcher (J.G.R.). The microcirculation was quantified as: Perfused Vessel Density (PVD), Percentage of Perfused Vessels (PPV), and Microvascular Flow Index (MFI, 0 = no flow, 1 = intermittent flow, 2 = sluggish flow, 3 = continuous flow [33,34]. *Bio-impedance analysis* was measured to assess body composition, using the phase sensitive Akern^®^ BIA 101 Anniversary edition (GLNP Life Sciences) with the patient in horizontal position using four electrodes: two on hand and two on the foot and an alternating current of 400 μA with a frequency of 50 kHz [35]. BIA measures Resistance (R), reflecting total body water, Reactance (Xc) reflecting membrane capacitance (cellular mass and integrity), and calculates Phase Angle (PA), the arctangent of Xc/R x 180/π, a marker of general health. Several routine parameters were collected: age, weight and height, routine markers of the circulation, fluid balance, lactate, norepinephrine dose and renal function. Furthermore, severity of disease was determined, using the routinely measured intensive care scores: Acute Physiology and Chronic Health Evaluation scores (APACHE III, APACHE IV) and the Sequential Organ Failure Assessment (SOFA) score [36,37]. At inclusion, renal function and damage were assessed by creatinine clearance measured from a 4-hours urine portion, urine output, urinary sodium and urinary albumin creatinine ratio. To determine whether patients developed AKI in the first week of ICU admission, both creatinine and urinary AKI criteria were determined daily during the first seven days of ICU admission. Pre-admission creatinine was defined as the last known creatinine value measured in disease free periods in the year before hospital admission and was retrieved from the hospital information system or from medical correspondence. When pre-admission serum creatinine value was not available, serum creatinine after recovery from critical illness was considered baseline value, when at least 2 stable values were available.

### Data analysis

Variables are presented as mean (SD), median (interquartile range) or number (percentage) as appropriate. All statistical analyses were performed using IBM SPSS Statistics 22^™^ (SPSS Inc., Chicago, IL, USA). We first compared the clinical characteristics and RRI of patients with and without AKI. For continuous variables, an independent T-test or Mann Whitney U test was used as appropriate and for nominal values a χ^2^-test was computed. To determine the predictive capacity of RRI for the development of AKI, potential predictors were first tested by univariate logistic regression analysis. Odds ratios were calculated. Subsequently, backward stepwise multivariable regression analysis (Wald) was computed to determine whether RRI remained an independent predictor of AKI when accounting for other risk factors, including significant variables as confounders with a maximum of n/10 variables [38]. A multivariate model was identified applying a p-entry and removal of less than 0.05. Collinearity and interactions were tested and the Hoshmer-Lemeshow test was used to check goodness-of-fit of the model. To determine discriminative value of the RRI alone and RRI in combination with other significant predictors of AKI, receiver operating characteristics (ROC) curves were constructed. The area under the curve (AUC) was determined with the optimal cut-off for sensitivity and specificity using Youden’s J statistic. P-values <0.05 were considered significant.

## Results

**Fig 1:**
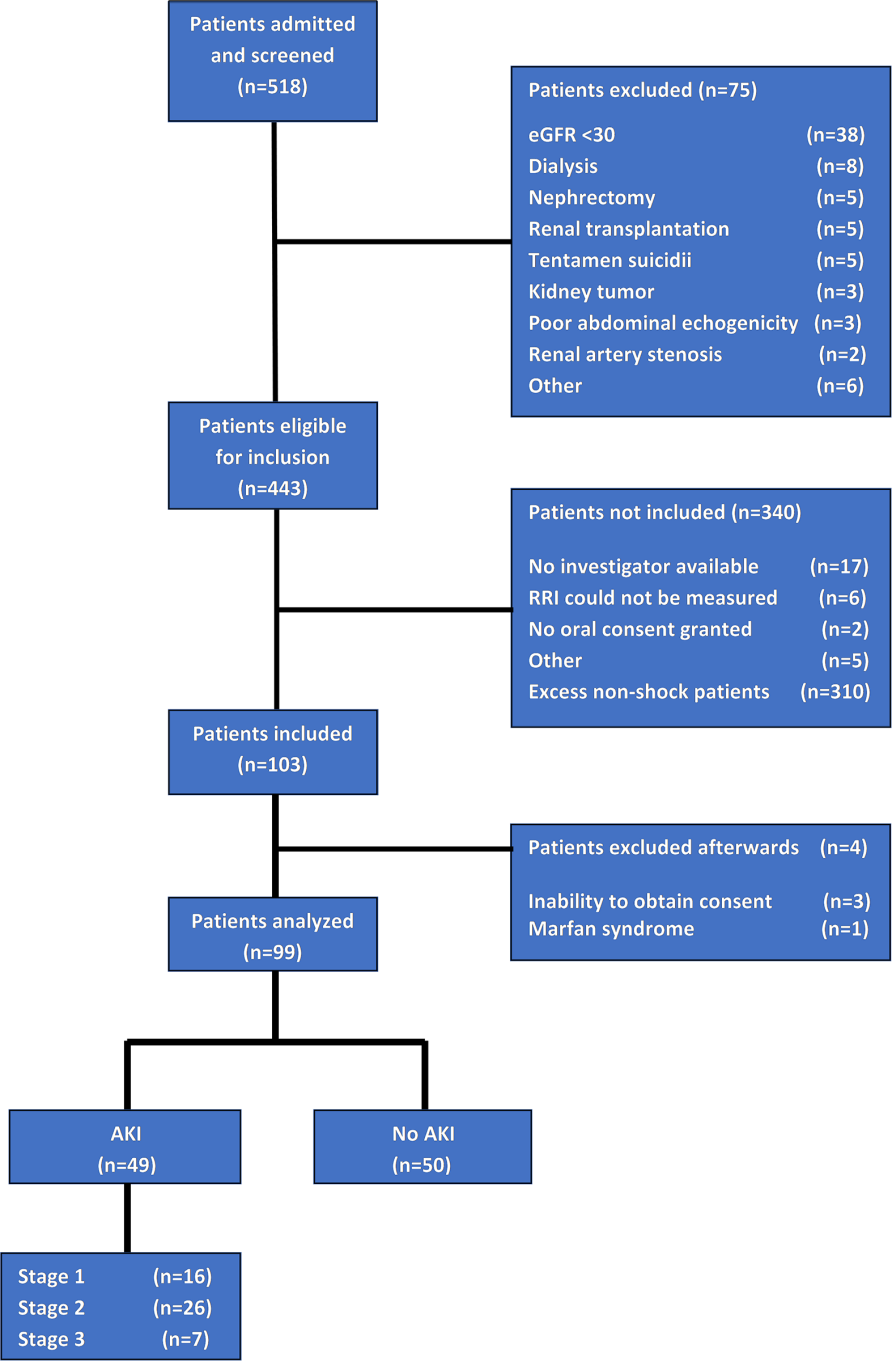
Flowchart diagram of inclusion.

From August 2015 until January 2016, 518 patients were admitted to the ICU. 75 patients were excluded due to chronic kidney conditions and other exclusion criteria, resulting in 443 patients eligible for inclusion. Three hundred ten non-shock patients were not measured because the planned number of inclusions of non-shock patients was completed. Thirty patients were not included due to a variety of reasons (Fig 1). One hundred three patients were included, three of whom were excluded from analysis, because deferred consent could not be obtained. One patient was excluded because his RRI was 0.397 due to connective tissue disease (Marfan syndrome).

A total of 99 patients were analyzed, 40 with shock and 59 without shock. Baseline characteristics of study patients are presented in Table 2 for patients with and without AKI separately. Baseline serum creatinine was not available in 21 patients. In these patients, we therefore used the last known stable serum value after ICU discharge. Forty-nine patients (49%) developed AKI within the first week of admission, 16 of whom suffered from stage 1 AKI, 26 from stage 2 AKI and 7 from stage 3 AKI. Patients developing AKI were older, had higher severity of disease scores, were more often in shock, received more often and higher amounts of vasopressors, had a more positive fluid balance, a lower phase angle and reactance and had a higher eGFR before admission and a lower creatinine clearance at inclusion (Table 2). Markers of the microcirculation on admission were not significantly different between patients developing AKI and those who did not.

**Table 2:** Comparison between baseline characteristics of patients with AKI and without AKI.

	Presented as	Patients with AKI (n = 49)	Patients without AKI (n = 50)	P-value
**Age in years**	Median (IQ range)	69 (60–78)	66 (55–71)	0.037
**Sex**				0.876
*Male*	*n (%)*	35 (71.4)	35 (70.0)	
*Female*	*n (%)*	14 (28.6)	15 (30.0)	
**BMI (kg/m**^**2**^**)**	Median (IQ range)	25.0 (23.1–28.4)	24.6 (21.7–27.9)	0.664
**ICU Severity Scores**				
*SOFA score at inclusion*	Mean (SD)	9.3 (5.8–12.8)	6.1 (3.8–8.4)	<0.001
*APACHE II score*	Mean (SD)	28 (18–38)	20 (19–22)	0.003
*APACHE III score*	Median (IQ range)	79 (64–107)	57 (45–70)	<0.001
**Types of admission**				
*Elective surgical*	*n (%)*	23 (46.9)	28 (56.0)	0.367
*Emergency surgical*	*n (%)*	6 (12.2)	6 (12.0)	0.970
*Medical*	*n (%)*	20 (40.8)	16 (32.0)	0.362
**Risk factors for AKI**				
*Pre-admission eGFR (ml/min)*^a^	Median (IQ range)	71 (64–86)	92 (70–100)	<0.001
* Moderate chronic kidney disease*^b^	*n (%)*	11 (22.4)	4 (8.0)	0.045
*Sepsis*	*n (%)*	7 (14.3)	4 (8.0)	0.320
*Hypertension*	*n (%)*	15 (30.6)	18 (36.0)	0.570
*Diabetes mellitus*	*n (%)*	8 (16.3)	9 (18.0)	0.825
**Organ function at inclusion**				
*Mean arterial pressure in mmHg*	Median (IQ range)	72 (65–82)	72 (67–86)	0.669
*Systolic blood pressure in mmHg*	Median (IQ range)	106 (99–123)	111 (94–128)	0.806
*Diastolic blood pressure in mmHg*	Median (IQ range)	56 (53–64)	57 (52–64)	0.843
*Heart rate in bpm*	Mean (SD)	81 (61–100)	77 (65–90)	0.353
*Central venous pressure in mmHg*	Median (IQ range)	7 (6–12)	6 (3–7)	0.017
*Cardiac output in L/min*	Median (IQ range)	4.1 (3.5–4.4)	3.6 (3.3–5.4)	0.920
*Central venous oxygen saturation in %*	Median (IQ range)	67 (64–73)	68 (64–75)	0.526
*Mechanical ventilation*	*n (%)*	48 (98.0)	45 (90.0)	0.097
*Fluid balance in mL*^c^	Median (IQ range)	1838 (521–4926)	553 (¯9–1355)	0.001
*Serum creatinine in μmol/L*	Mean (SD)	96 (61–131)	70 (55–84)	<0.001
*Serum urea in in mmol/L*	Median (IQ range)	7.0 (6.0–9.0)	6.0 (5.0–7.0)	0.264
*Urine micro-albumin in mg/L*	Median (IQ range)	21 (8–54)	12 (5–25)	0.026
*Albumin/creatinine ratio*	Median (IQ range)	4.27 (1.10–10.25)	2.65 (1.71–4.72)	0.107
*Urine sodium in mmol/24h*	Median (IQ range)	21 (<20–75)	90 (46–112)	0.001
*Creatinine clearance in ml/min/1*.*73 m*^*2*^ ^d^	Median (IQ range)	53 (29–103)	107 (75–134)	<0.001
**Treatment at inclusion**				
*Patients receiving norepinephrine*	*n (%)*	30 (61.2)	18 (36.0)	0.012
*Norepinephrine support during measurement in mg/kg/h*	Median (IQ range)	0.72 (0.0–1.4)	0.0 (0.0–0.7)	0.001
*Patients receiving nephrotoxic medication*^e^	*n (%)*	6 (12.2)	5 (10.0)	0.722
**Outcome**				
*Length of Stay in days*	Median (IQ range)	5 (3–12)	2 (2–3)	<0.001
*28 day all-cause mortality*	n (%)	9 (18.4)	3 (6.0)	0.059

BMI: Body Mass Index (kg/m^2^). SOFA score: Sepsis-related Organ Failure Assessment score. APACHE: Acute Physiology and Chronic Health Evaluation

^a^ eGFR was calculated using the CKD-EPI formula

^b^ Chronic kidney disease was defined as an eGFR before ICU admission of 30–60 mL/min, patients with an eGFR < 30 ml were excluded

^c^ Fluid balance at inclusion starting from ICU admission until measurements (<24h after admission)

^d^ Creatinine clearance (4 hours portion)

^e^ e.g. aminoglycosides, vancomycin, aciclovir, chemotherapeutics, NSAIDs etc. Including radiocontrast

### Primary outcome

**Fig 2:**
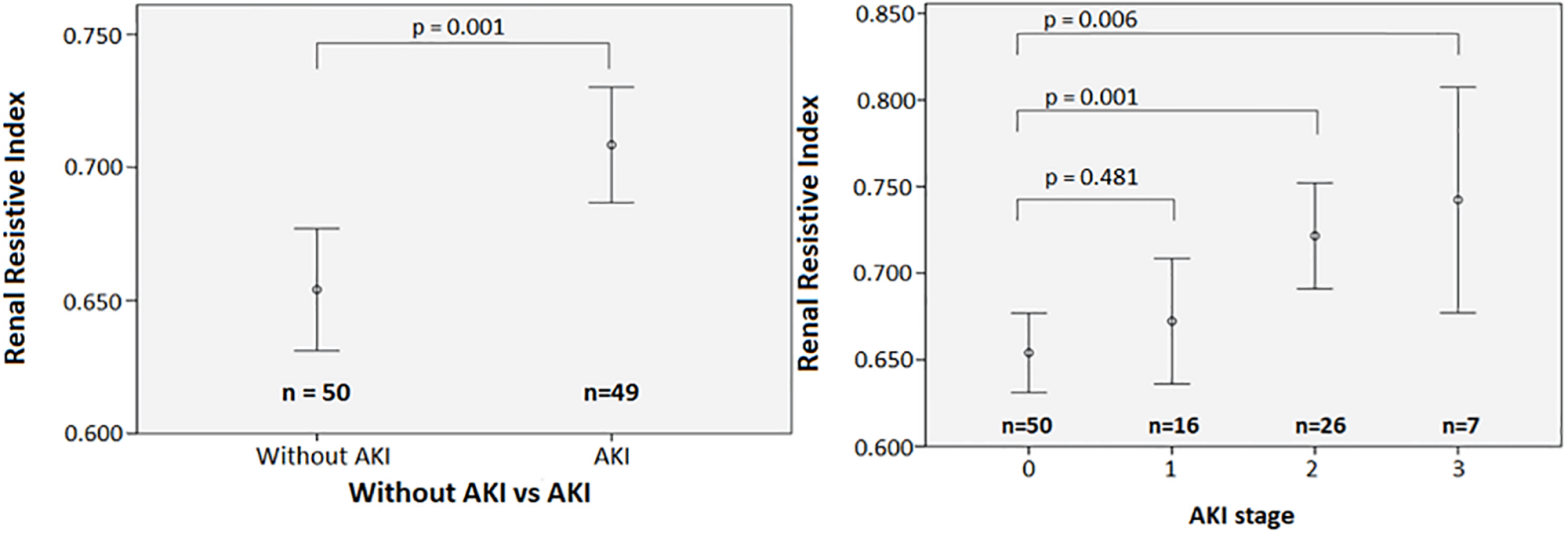
Renal resistive index (mean, 95% CI) for patients without AKI and patients developing AKI (A) renal resistive index (mean, 95% CI) for the different stages of AKI (B).

RRI averages could be calculated for both kidneys in 97 patients and for one kidney in 2 patients. The values from left and right kidneys were averaged because there was no significant difference between left and right (means: 0.682 vs. 0.681, difference 0.07%, p = 0.796). RRI on admission was 0.71 (95% CI 0.69–0.73) in patients developing AKI and 0.65 (95% CI 0.63–0.68) in patients not developing AKI (p = 0.001) (Table 3 and Fig 2).

**Table 3:** Study parameters.

	Presented as	Patients with AKI	Patients without AKI	P-value
**Renal Resistive Index (RRI)**		**(n = 49)**	**(n = 50)**	
*Both kidneys averaged*	Mean (95% CI)	0.708 (0.687–0.730)	0.654 (0.631–0.677)	0.001
*Left kidney*	Mean (95% CI)	0.706 (0.683–0.730)	0.657 (0.632–0.682)	0.005
*Right kidney*	Mean (95% CI)	0.711 (0.690–0.732)	0.652 (0.629–0.675)	<0.001
**Microcirculation (SDF)**		**(n = 36)**	**(n = 33)**	
*Vessel Density (n/mm)*	Mean (95% CI)	9.96 (9.32–10.61)	9.11 (8.23–9.99)	0.110
*Percentage of Perfused Vessels (%)*	Mean (95% CI)	73.6 (69.4–77.7)	72.9 (67.9–78.0)	0.146
*Perfused Vessel Density (n/mm)*	Mean (95% CI)	7.32 (6.73–7.92)	6.65 (5.93–7.37)	0.841
*Microvascular Flow Index*	Mean (95% CI)	1.97 (1.79–2.14)	2.05 (1.87–2.24)	0.482
**Bioelectral Impedance Analysis (BIA)**		**(n = 47)**	**(n = 49)**	
*Resistance (Xc/m)*	Median (IQ range)	276.9 (246.8–310.7)	285.0 (255.4–322.1)	0.631
*Reactance (R/m)*	Median (IQ range)	22.1 (17.4–27.8)	24.7 (22.8–31.1)	0.015
*Phase Angle*	Median (IQ range)	4.3 (3.6–5.4)	5.1 (4.4–6.2)	0.010

### Secondary outcome

RRI was significantly higher in patients developing AKI stage 2 (RRI = 0.72, 95% CI 0.65–0.80) and 3 (RRI = 0.74, 95% CI 0.67–0.81) than in patients without AKI (RRI = 0.65, 95% CI 0.63–0.68) p = 0.001 and 0.006 respectively (Fig 2). RRI was not significantly different between AKI stage 1 (RRI: 0.67, 95% CI 0.61–0.74) and non-AKI (p = 0.481).

### Discrimination of AKI

**Fig 3:**
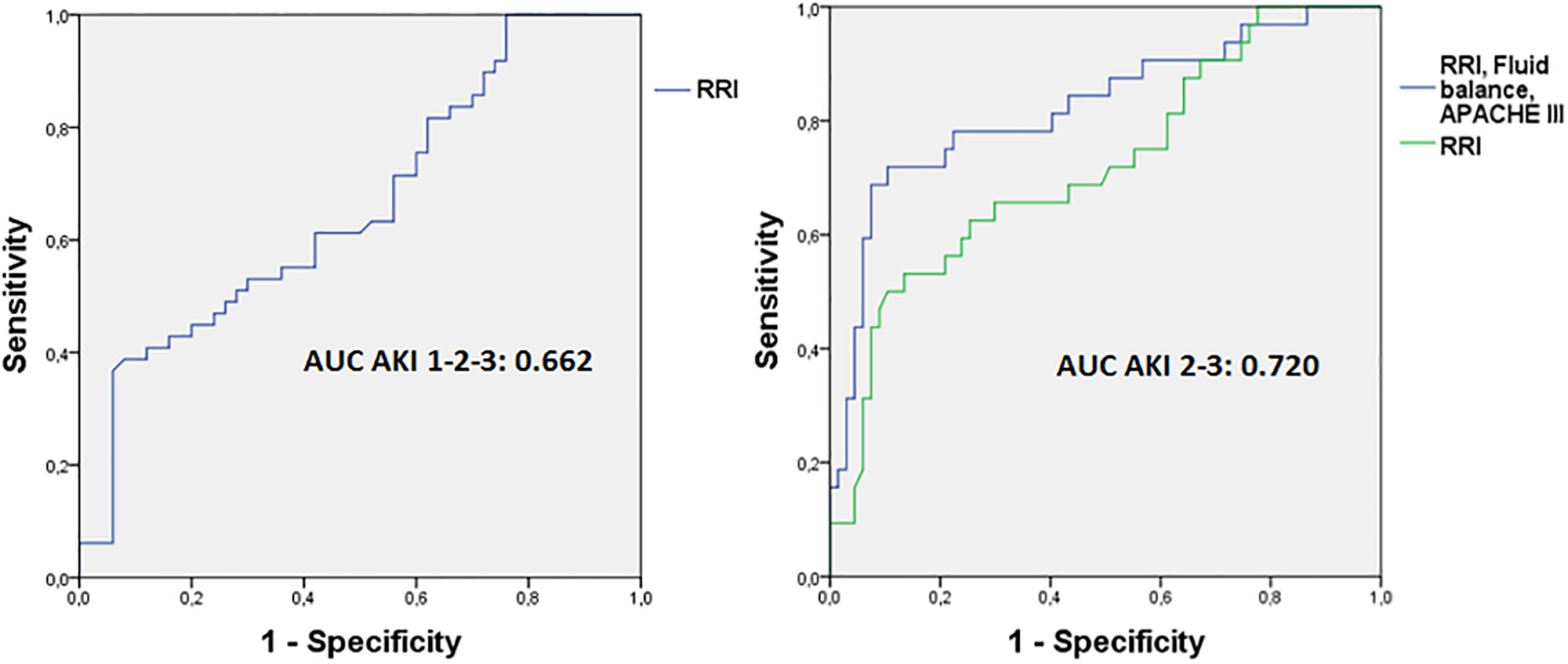
ROC curve of RRI for AKI stage 1,2 and 3 (A) ROC curve for AKI stage 2 and 3: RRI and RRI combined with APACHE III and fluid balance. (A): RRI; AUC 0.662, (95% CI 0.556–0.769), SE 0.055, p = 0.005 (B), green line: RRI; AUC 0.720, (95% CI 0.612–0.831), SE 0.056, p = <0.001 (B), blue line: RRI plus Fluid balance plus APACHE III; AUC 0.825, (95% CI 0.732–0.921), SE 0.048, p = <0.001.

The ROC curve of RRI on admission (Fig 3) showed a poor discrimination of AKI development during the first week versus non-AKI (AUC of 0.66, 95% CI 0.56–0.77). The AUC of the ROC curves of pre-admission serum creatinine, fluid balance, and MAP showed poor discrimination for AKI (AUC <0.7) for each of the variables separately as well, with fluid balance being the best discriminating variable (AUC = 0.70). Since RRI was not a good discriminator of AKI stage 1, and patients with AKI stage 2 and 3 had a significantly higher RRI compared to non-AKI, we continued the analysis with AKI stages 2 and 3 combined (Fig 3). The AUC of RRI to predict AKI stage 2 or 3 was 0.72 (95% CI 0.61–0.83) and for AKI stage 3 0.74 (95% CI 0.55–0.93). However, the number of inclusions for stage 3 (n = 7) was small. The optimal cut-off point of RRI for discrimination of AKI stage 2–3 was 0.74, with a sensitivity and specificity of respectively 53% and 87%.

### Prediction of AKI stage 2 and 3, univariate analysis

Univariate analysis (Table 4) revealed that apart from RRI, APACHE III score, age, CVP, norepinephrine dose, pre-admission creatinine, fluid balance, reactance and phase angle, fluid balance were significant predictors of AKI stage 2–3.

**Table 4:** Univariate regression analysis for AKI stage 2 and 3.

	n	OR	OR (95%CI)	P-value
**Study parameters**				
*Renal Resistive Index*	99	1.012	1.006–1.019	<0.001
*Vessel Density (n/mm)*	69	1.145	0.908–1.443	0.253
*Perfused Vessel Density (n/mm)*	69	1.075	0.828–1.394	0.588
*Percentage of Perfused Vessel (%)*	69	0.989	0.952–1.027	0.568
*Microvascular Flow Index (MFI)*	69	0.580	0.223–1.513	0.266
*Resistance (R/m)*	96	0.997	0.990–1.005	0.490
*Reactance (Xc/m)*	96	0.918	0.861–0.978	0.008
*Phase Angle*	96	0.586	0.399–0.861	0.007
**Systemic circulation**				
*Mean arterial pressure (mmHg)*	99	0.969	0.930–1.009	0.125
*Systolic blood pressure (mmHg)*	99	0.983	0.961–1.005	0.123
*Diastolic blood pressure (mmHg)*	99	0.978	0.932–1.027	0.380
*Heart rate (bpm)*	99	1.021	0.994–1.048	0.134
*Mixed venous oxygen saturation*	66	0.943	0.883–1.007	0.081
*Cardiac Output*	31	0.809	0.434–1.506	0.504
*Central venous pressure*	43	1.202	1.014–1.425	0.034
*Vasopressor support*	99	0.233	0.093–0.582	0.002
*Norepinephrine dose (mL/kg/h)*^a^	99	1.259	1.110–1.428	<0.001
**Hydration state**				
*Fluid balance (L)*^b^	99	1.530	1.242–1.885	0.001
*Diuresis (L)*	99	0.718	0.422–1.222	0.222
**Renal function at inclusion**				
*Creatinine clearance (ml/min/1*.*73m*^*2*^*)*^c^	98	0.967	0.953–0.982	<0.001
*Urea (mmol/24h)*	97	0.997	0.992–1.002	0.248
*Sodium (mmol/24h)*	95	0.976	0.962–0.989	0.001
*Micro-albumin (mg/24h)*	98	1.001	0.998–1.004	0.441
*Albumin/Creatinine ratio*	98	1.015	0.996–1.034	0.133
**Other**				
*Age*	99	1.038	1.002–1.076	0.041
*APACHE III score*	99	1.031	1.015–1.048	<0.001
*Pre-admission creatinine (μmol/L)*	99	1.046	1.019–1.073	0.001
*BMI (kg/m*^*2*^*)*	99	1.068	0.967–1.179	0.196
*Use of nephrotoxic drugs*^d^	99	0.531	0.149–1.892	0.329
* Diabetes*	99	0.466	0.161–1.350	0.159

APACHE: Acute Physiology and Chronic Health Evaluation. BMI: Body Mass Index (kg/m^2^)

^a^ Norepinephrine dose at times of RRI measurement

^b^ Fluid balance at inclusion starting from ICU admission until measurements (<24h after admission)

^c^ Calculated through a 4-hour urine portion

^d^ e.g. aminoglycosides, vancomycin, aciclovir, chemotherapeutics, NSAIDs etc. Including radiocontrast

### Prediction of AKI stage 2–3, multivariate analysis

To determine whether RRI was an independent predictor of AKI, the five most significant variables in univariate analysis were analyzed as confounders for AKI prediction in multivariate analysis in addition to RRI: norepinephrine support, urinary sodium, fluid balance, phase angle and APACHE III as global marker of severity of illness. When adding these variables, RRI remained a predictor of AKI, independent of APACHE III score and fluid balance. Phase angle, urinary sodium and norepinephrine dose were removed as being not significant anymore (Table 5). The R^2^ of the model was 0.426, the Hosmer Lemeshow goodness-of-fit chi square test was 8.008 (df = 8, p = 0.433). There were no multi-collinearity issues or significant interactions. The ROC curve of this model had an AUC of 0.84 (95% CI 0.75–0.93) (Fig 3), sensitivity 80.0% specificity 71.4%, positive predictive value: 73.7%, negative predictive value: 78.1%.

**Table 5:** Multivariate regression analysis for AKI stage 2 and 3.

	Odds-ratio	95%CI	P-value
*Renal Resistive Index*	1.008	1.000–1.016	0.043
*APACHE III*	1.021	1.004–1.039	0.013
*Fluid balance (L)*	1.348	1.091–1.666	0.006
**Variables included:**	RRI, APACHE III, norepinephrine dose, fluid balance, phase angle, urinary sodium		
**Variables removed:**	Step 2: Phase angle, step 3: Norepinephrine dose (mL/kg/h), step 4: Urinary sodium mmol/24h		
**n**	93		
**Hosmer Lemeshow on step 4**	8.008 (df = 8, p = 0.433)		
**Nagelkerke R**^**2**^ **on step 4**	0.426		

Pre-admission eGFR and serum creatinine were not included in the multivariate analysis. Reason was that eGFR is determined by preadmission creatinine and that the ratio of preadmission/admission creatinine classifies the patients as having AKI (the dependent variable). The dependent an independent variable are therefore mathematically coupled. Moreover, serum creatinine is already reflected in the APACHE III score. However, since pre-admission preadmission eGFR is one of the strongest predictors of AKI, a model including pre-admission eGFR is available in the electronic supplement. When including eGFR, RRI was not a significant predictor anymore (see e-supplement).

## Discussion

This prospective observational study shows that RRI on ICU admission was a significant independent early predictor and discriminator for development of AKI stage 2 and 3 during the first week, but not for AKI stage 1. The optimal cut-off point of RRI for discrimination of AKI stage 2–3 in our population was 0.74 (sensitivity 53%, specificity 87%). Prediction of AKI increased when severity of disease (APACHE III) and fluid balance were taken into account. This indicates that RRI alone is not sensitive enough to be used as a solitary discriminator for developing AKI. However if RRI is high, the risk of developing AKI is high especially in patients with a high severity of disease and a positive fluid balance. Since early predictors of AKI are not available yet, measuring RRI as part of a broad ultrasound screening of vital organ function could contribute to the early detection of patients at risk of developing AKI and trigger measures to protect the kidney such as circulatory optimization and limiting exposure to other risk factors of AKI.

### Interpretation of RRI and its cut-off value

RRI was higher in patients developing AKI than in those who did not. This finding is consistent with most studies in a mixed ICU population, patients with septic shock and after cardiac surgery [21,27,28,39,40]. However, absolute values differed and there was overlap in some studies [22,41,42]. In addition, RRI values were higher in AKI stage 2–3 but not in AKI stage 1 patients. This finding is also consistent with the literature: higher values are reported in patients with more severe or persistent AKI [21,28,39,40,43–47]. A recent meta-analysis showed that high RRI was a good predictor for persistent, but not for transient AKI [47]. This is in line with the results of the present study, namely that RRI is a significant predictor for the more severe stages of AKI (2 and 3) but not for stage 1. The study performed by Lerolle et al. shows a similar result in patients with septic shock [21]. In Lerolle’s study RRI on ICU admission was predictive for stage I (Injury) and F (Failure) according to the RIFLE classification, but not for stage R (Risk). These results suggest that RRI may be related to the severity of renal dysfunction. The cut-off value of for AKI stage 2–3 in our population was 0.74. Cut-off values reported in previous studies in different ICU populations vary ance and APACHE scoreted in fluid baIleby mdrd or higher than pre-admission due to persistent loss of renal function. die late between 0.71 and 0.80 [21,27,28,43,48–50], while normal values are about 0.60 with an upper limit of 0.70. Cut-off values depend on the definition of AKI. The use of both creatinine and urine criteria to diagnose AKI as we did increases the proportion of patients diagnosed with AKI and will yield lower cut-off values than when only creatinine criteria are used. Cut-off values are also population specific and depend on the prevalence of AKI in the population of study. We included a mixed intensive care population with a high proportion of shock patients. Previous studies included other populations: major thoracic or orthopedic surgery [27,40,43,48,49], coronary angiography with contrast [51], mechanical ventilation [28], and septic shock [21]. One study evaluated the RRI in patients who had already developed AKI or prerenal azotemia [49]. The lowest cut-off variable was established in patients in the recovery room after orthopedic surgery [50], whereas the highest cut-off point was found in a mixed ICU population for persistent AKI [28]. A high cut-off value (0.85) has also been reported in established AKI and being predictive of non-recovery [52]. Thus, RRI seems a marker for the severity of AKI.

### RRI in the context of other predictors

In our population, RRI alone appeared as a moderate predictor of AKI. The relationship between RRI and the development of AKI likely reflects multiple pathogenic pathways, including prerenal, intrarenal and postrenal factors. Intrarenal vasoconstriction in response to shock or poor pre-existent compliance of the renal vessels [53–55], reflecting acute on chronic endothelial dysfunction [56], or renal outflow impediment (high CVP) might play a role in our patients. RRI as a sole predictor, must be interpreted with caution, as the determinants of AKI are multiple and most remain undiscovered [7,20,54,57–59]. Other factors than vasoconstriction or poor vessel compliance contribute to AKI as well [60,61]. Severity of illness improved the prediction of AKI in our population and this finding is in line with previous RRI studies [62]. However, our study is the first study accounting for changes in the microcirculation, fluid balance and body composition. Despite the role of renal microcirculatory alterations in AKI [5,6], sublingual microcirculation on admission was not predictive of AKI development. This may be due to heterogeneity of the microcirculation [32], and therefore sublingual microcirculation may not reflect renal microcirculation. Reason to include BIA was that BIA provides an objective measure of fluid status (resistance) and membrane capacitance (reactance), and calculates phase angle, a marker of cellular vitality which is independently related to mortality in ICU patients [63]. Resistance was not predictive of AKI, but reactance and phase angle were significantly related to the development of AKI on univariate analysis. However, both were excluded on multivariate analysis. They were apparently represented by fluid balance and APACHE. The independent prediction of fluid balance is interesting. A positive fluid balance may be a consequence but also a cause of AKI by decreasing renal perfusion due to interstitial edema and or higher CVP [55,64]. In case of capillary leakage, part of the administered fluid enters the tissues, resulting in tissue edema and thereby reducing tissue perfusion. CVP was related to RRI, but the measurement was not among the most significant ones and only available in 43% of the patients and therefore not included in the multivariate analysis. Diastolic blood pressure was not significantly related to the development of AKI. This finding is noteworthy, for diastolic blood pressure has been shown to be significantly lower in patients with AKI in a previous study [55]. Although pre-existent renal dysfunction is an important risk factor for AKI, we did not include eGFR in the model because of the mathematical relation between eGFR and AKI classification. Moreover, eGFR is often estimated because preadmission creatinine is not always available, while RRI is available at the bedside. Altogether, with an AUC of 0.83, the discriminative performance of our day-1 AKI 2–3 prediction model, including three predictors namely RRI, severity of disease and fluid balance, was as good as the recently published day 1 AKI prediction model from the Leuven group which had an AUC of 0.84 and included 13 clinically available predictors [65]. However, our study was not powered to develop a prediction model but rather to determine whether RRI could help to predict AKI development in the light of other risk factors.

Our study has several strengths and limitations. The present study is the first study determining the predictive and discriminative value of RRI on ICU admission for the development of AKI at any point during the first week in a mixed medical/surgical ICU population, accounting for changes in the microcirculation, fluid balance and body composition. Up to now, it is the largest study examining the predictive value of RRI for the development of AKI in any ICU population [47]. Furthermore, we used both urine and creatinine criteria for AKI diagnosis, while some previous studies used creatinine criteria alone [27,48,49,51]. In addition, final RRI was the calculated from 9 measurements taken from two kidneys. Another strength is that two investigators included all patients and performed all measurements together, increasing the reliability of the results. Moreover, all measurements were performed in a short time frame. Finally, we included a large heterogeneous ICU population, which increases the generalizability. A possible limitation of this study is the high proportion of shock in our population. To increase the hazard of AKI we decided to include at least 40 patients with shock. This should be taken into account when transposing our findings to the general ICU population. Second, the observational design of the study prohibits showing a causal relation between RRI and the development of AKI. We only showed an association and earlier prediction of AKI than the use of creatinine-based definition of AKI. Third, we did not include CVP in the multivariate analysis since inclusion would be associated with loss of data. Additionally, CVP was not amongst the most significant variables. A further limitation was that patients admitted during the weekend, were not included because the investigators were not available. However, the severity of illness in this non-included population was similar (data not shown). Ultimately, a pre-admission creatinine was not available in 21% of the patients, challenging the diagnosis of AKI. This is a well-known pitfall in all studies on AKI. To estimate baseline serum creatinine, we used the latest known value after at least 2 steady state creatinine values to calculate pre-admission eGFR as done in previous studies [28,47]. This method may be criticized because creatinine values may be falsely low due to muscle wasting or higher than pre-admission due to incomplete renal recovery. Another solution would be to estimate baseline serum creatinine by the MDRD equation. This method appeared acceptable when pre-morbid GFR was near normal, but overestimated the incidence of AKI for patients with chronic kidney disease [66].

## Conclusions

In this prospective observational study in a mixed ICU population, high RRI on ICU admission was a significant independent early predictor for development of AKI stage 2–3 during the first week, but not for AKI stage 1. Although RRI alone was not a sensitive discriminator for developing AKI, high RRI can be used as an early warning signal in the setting of a high severity of disease and a positive fluid balance. RRI appeared as a predictor of AKI, independent of APACHE III and fluid balance, suggesting that that intrarenal vasoconstriction or poor vascular compliance, severity of disease and positive fluid balance independently contribute to the development of AKI. Large multicenter studies are needed to confirm whether the patients with RRI, as identified during the routine ultrasound screening of vital organ function on ICU admission, will benefit from interventions aiming to prevent AKI and whether RRI is useful to monitor interventions such vasopressor support.

## Supporting information

S1 Fig ROC curves of seven most significant univariate predictors of AKI stage 2 and 3. (TIF)

S1 Table Multivariate regression analysis for AKI stage 2 and 3 including pre-admission eGFR. Chronic renal insufficiency is an important risk factor for AKI. On request of the reviewer, we therefore performed an additional multivariate analysis including pre-admission eGFR (as measured with the CKD EPI method) in addition to the most significant other variables. In this analysis, norepinephrine dose and urinary sodium remained as significant predictors. None of the predictors from the original model (APACHE III score, fluid balance and RRI) remained significant once pre-admission eGFR was added. The reason why we did not include eGFR in primary multivariate analysis is that calculated eGFR and the definition of AKI are mathematically coupled. Pre-admission eGFR is determined by preadmission creatinine, and AKI diagnosis is based on the ratio of creatinine during admission and pre-admission creatinine. Moreover, eGFR is often not available, as was the case in 21% of the patients included in this study. In these patients, eGFR was estimated based on steady state creatinine after discharge from the ICU.
(DOCX)

## Abbreviations

AKI: Acute Kidney Injury
APACHE: Acute Physiology and Chronic Health Evaluation
BIA: Bioelectral Impedance Analysis
CI: Confidence Interval
CVP: Central Venous Pressure
eGFR: Estimated Glomerular Filtration Rate
HI: Heterogeneity Index
ICU: Intensive Care Unit
IQR: Interquartile Range
KDIGO: Kidney Disease Improving Global Outcomes
MAP: Mean Arterial Pressure
MFI: Microvascular Flow Index
PPV: Proportion of Perfused Vessels
PVD: Perfused Vessel Density
RRI: Renal Resistive Index
ROC: Receiver Operating Characteristics
SDF: Sidestream Dark Field
SIRS: Systemic Inflammatory Response Syndrome
SOFA: Sepsis related Organ Failure Assessment
SvO_2_: Venous Oxygen Saturation
VD: Vessel Density
