# Supplementary figures and images for: Renal resistive index as an early predictor and discriminator of acute kidney injury in critically ill patients; A prospective observational cohort study
Source: PLoS One. 2018 Jun 11;13(6):e0197967. doi: 10.1371/journal.pone.0197967 (PMC5995360; doi:10.1371/journal.pone.0197967)

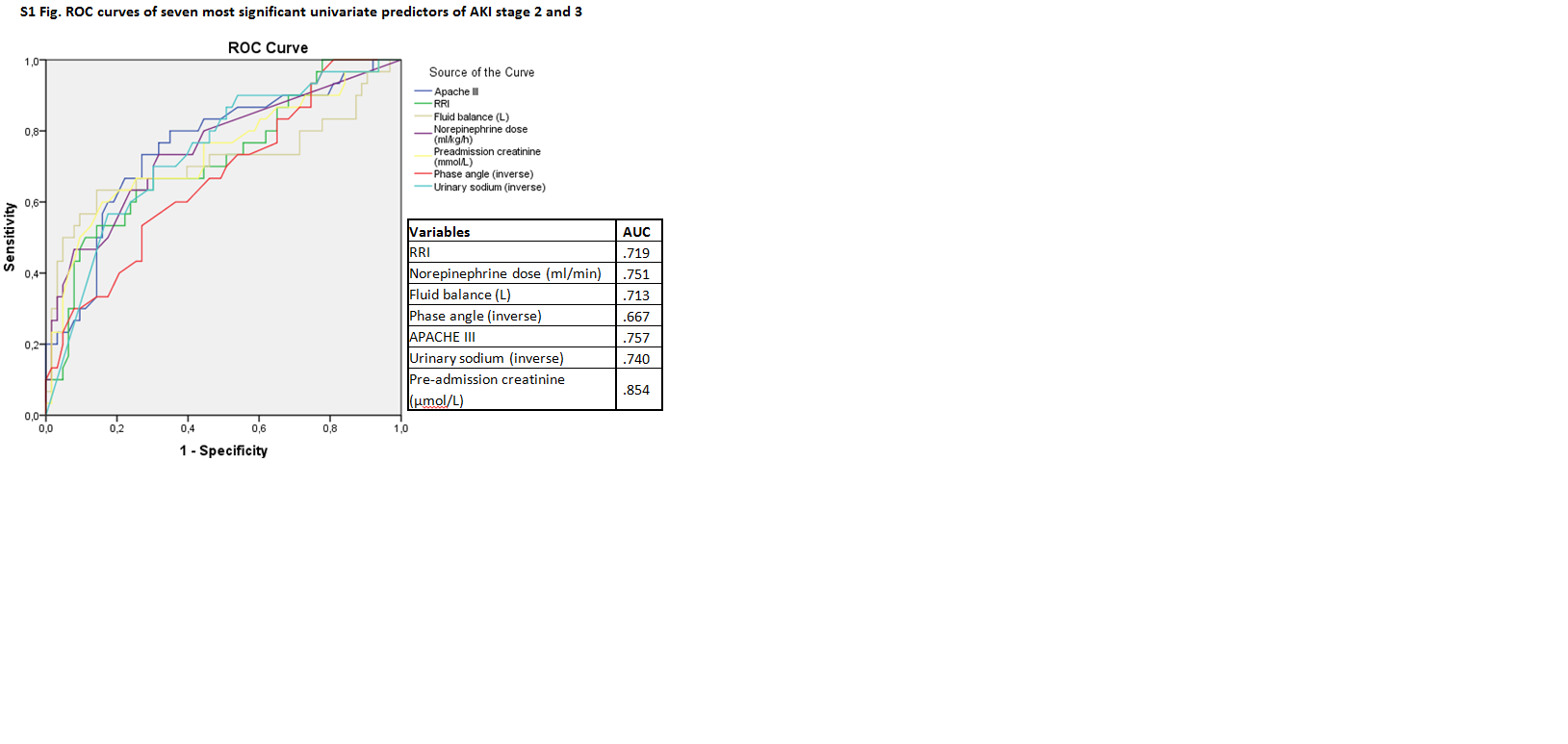

Supplement: S1 Fig — (TIF) [file pone.0197967.s001.tif]
