# Supplementary material for: Renal resistive index as an early predictor and discriminator of acute kidney injury in critically ill patients; A prospective observational cohort study
Source: PLoS One. 2018 Jun 11;13(6):e0197967. doi: 10.1371/journal.pone.0197967 (PMC5995360; doi:10.1371/journal.pone.0197967)
Supplement: S1 Table — Chronic renal insufficiency is an important risk factor for AKI. On request of the reviewer, we therefore performed an additional multivariate analysis including pre-admission eGFR (as measured with the CKD EPI method) in addition to the most significant other variables. In this analysis, norepinephrine dose and urinary sodium remained as significant predictors. None of the predictors from the original model (APACHE III score, fluid balance and RRI) remained significant once pre-admission eGFR was added. The reason why we did not include eGFR in primary multivariate analysis is that calculated eGFR and the definition of AKI are mathematically coupled. Pre-admission eGFR is determined by preadmission creatinine, and AKI diagnosis is based on the ratio of creatinine during admission and pre-admission creatinine. Moreover, eGFR is often not available, as was the case in 21% of the patients included in this study. In these patients, eGFR was estimated based on steady state creatinine after discharge from the ICU. (DOCX) [file pone.0197967.s002.docx]

**S1 Table. Multivariate regression analysis for AKI stage 2 and 3 including pre-admission eGFR**

|  | | **Odds-ratio** | **95%CI** | **P-value** |
| --- | --- | --- | --- | --- |
| Pre-admission eGFR (ml/min) | | 0.935 | 0.903-0.969 | <0.001 |
| Norepinephrine dose (mL/kg/h) | | 1.269 | 1.077-1.495 | 0.004 |
| Urinary sodium mmol/24h | | 0.977 | 0.960-0.995 | 0.014 |
| **Variables included:**  **Variables removed:** |  | RRI, APACHE III, norepinephrine dose, fluid balance, phase angle, urinary sodium, pre-admission eGFR  Step 2: Phase angle, step 3: APACHE III, step 4: *Renal Resistive Index*, step 5: Fluid balance (L) | | |
| **n** |  | 93 | | |
| **Hosmer Lemeshow on step 5** |  | 9.847 (df=8, p=0.276) | | |
| **Nagelkerke R^2^ on step 4** |  | 0.557 | | |

Chronic renal insufficiency is an important risk factor for AKI. On request of the reviewer, we therefore performed an additional multivariate analysis including pre-admission eGFR (as measured with the CKD EPI method) in addition to the most significant other variables. In this analysis, norepinephrine dose and urinary sodium remained as significant predictors. None of the predictors from the original model (APACHE III score, fluid balance and RRI) remained significant once pre-admission eGFR was added. The reason why we did not include eGFR in primary multivariate analysis is that calculated eGFR and the definition of AKI are mathematically coupled. Pre-admission eGFR is determined by preadmission creatinine, and AKI diagnosis is based on the ratio of creatinine during admission and pre-admission creatinine. Moreover, eGFR is often not available, as was the case in 21% of the patients included in this study. In these patients, eGFR was estimated based on steady state creatinine after discharge from the ICU.
